# Exploring nutrient intake and Mediterranean diet adherence across BMI categories in a Spanish adult population

**DOI:** 10.3389/fnut.2025.1689298

**Published:** 2025-12-12

**Authors:** Iciar Martin-Llaguno, Marcelo Saval-Calvo, Maria Florez-Martin, Laura Martin-Manchado, Ana Zaragoza-Marti

**Affiliations:** 1 Department of Analytical Chemistry, Nutrition and Bromatology, University of Alicante, Sant Vicent del Raspeig, Spain; 2 Department of Computer Technology, University of Alicante, Sant Vicent del Raspeig, Spain; 3 Division of Surgery and Interventional Sciences, University College London, London, United Kingdom; 4 Department of Nursing, Faculty of Health Sciences, University of Alicante, Sant Vicent del Raspeig, Spain; 5 Alicante Institute for Health and Biomedical Research (ISABIAL), Alicante, Spain

**Keywords:** dietary compliance, nutritional deficiencies, nutritional adequacy, obesity, Mediterranean diet

## Abstract

**Introduction:**

Obesity is often associated with excessive energy intake, yet individuals with obesity may also present with micronutrient inadequacies. This study examined dietary compliance and nutrient adequacy across Body Mass Index (BMI) categories in a sample of Spanish adults living in a Mediterranean context, and explored how adherence to the Mediterranean diet relates to BMI.

**Methods:**

A cross-sectional study was conducted between September 2020 and June 2021 among 167 adults classified as normal weight (*n* = 38), overweight (*n* = 47), or obese (*n* = 82). Participants meeting exclusion criteria such as pregnancy, endocrine disorders, or psychiatric illness were omitted. Dietary intake was assessed using a validated food frequency questionnaire specific to the Mediterranean population, complemented by 3-day dietary records. Nutrient adequacy was determined using European Food Safety Authority (EFSA) Dietary Reference Values, and adherence to the Mediterranean diet was measured with the 14-item MEDAS questionnaire.

**Results:**

Vitamin D and iodine inadequacy were highly prevalent across all BMI groups (96.41% and 74.85%, respectively). While unadjusted analyses suggested lower intakes of fiber, potassium, zinc, folate, and vitamins A, B1, C, and E in obese compared with normal-weight participants, these differences were not statistically significant after correction for multiple comparisons (false discovery rate *q* = 0.05). Small effect sizes (ε^2^ = 0.03–0.05) indicated consistent but modest trends toward lower micronutrient intake with increasing BMI. Average energy intake exceeded recommendations in all groups. Higher adherence to the Mediterranean diet was more common among normal-weight individuals and was inversely associated with BMI after adjusting for sex, educational level, labor status, physical activity, and energy intake.

**Discussion:**

In this Mediterranean sample, obesity was not explained by total energy intake alone. Although differences in specific nutrient intakes did not remain statistically significant after adjustment, trends suggest lower dietary quality among individuals with obesity. Promoting nutrient-dense dietary patterns such as the Mediterranean diet may support healthier weight status.

## 1 Introduction

Obesity is a chronic, multifactorial disease characterized by excessive adipose tissue accumulation, recently reaffirmed as such by the World Health Organization (WHO) (1). Its prevalence has risen sharply over the past three decades, with more than 1.9 billion adults currently classified as overweight and over 650 million as obese (2–4). In Europe, overweight and obesity have reached epidemic proportions, affecting nearly 60% of adults in the WHO European Region (1). Projections suggest that obesity prevalence will peak between 2026 and 2054, with the United States and the United Kingdom expected to reach the highest levels first, followed by similar trends across other European nations (5). Comparable upward trends have also been observed in Asia and in the Middle East and North Africa (MENA) region, where obesity prevalence has risen rapidly in recent decades, reaching some of the highest levels worldwide (6–8). In Spain, national data from 2016 reported an obesity prevalence of 21.6% among adults (9, 10), and estimates from the Organization for Economic Co-operation and Development (OECD) indicated that 53% of the population was overweight (11). Forecasts predict a further 16% increase in the overweight population by 2030 (12).

Obesity is a well-established risk factor for numerous non-communicable diseases (13, 14), including type 2 diabetes (15), hypertension, cardiovascular disease (16–19), stroke and transient ischemic attack (20), musculoskeletal disorders, and several cancers (16, 18, 21). Its population-level impact extends beyond morbidity: overweight and obesity account for roughly 7% of total years lived with disability (YLDs) in the WHO European Region (1), with comparable global patterns (22, 23). Economically, obesity imposes a substantial burden on health systems. In an analysis of 161 countries, overweight and obesity represented a major share of healthcare expenditure, largely due to increased inpatient, outpatient, and medication costs compared with normal-weight individuals (24).

Alongside these health and economic consequences, obesity increasingly exemplifies a paradox of excessive energy intake combined with nutritional inadequacy. Diets rich in ultra-processed, energy-dense foods often provide surplus calories while remaining poor in essential vitamins and minerals. Moreover, obesity-related metabolic changes, including chronic low-grade inflammation and altered storage or bioavailability of fat-soluble micronutrients, may further impair nutrient utilization. These mechanisms help explain why individuals with obesity can present both overnutrition and micronutrient deficiencies.

Although a sustained imbalance between energy intake and expenditure remains a primary driver of weight gain, obesity is now recognized as an independent contributor to cardiometabolic disease risk (1, 25). Increasing evidence also highlights the importance of diet quality in obesity development and progression. Individuals with obesity frequently exhibit suboptimal macronutrient profiles and inadequate micronutrient intake despite high caloric consumption (26–30). Empirical studies from Spain, Chile, France, and the United States have reported deficiencies in folate, vitamins D, B12, A, C, and E, as well as minerals such as iron, zinc, magnesium, calcium, and copper among adults with obesity (30–33). These inadequacies may arise from dietary patterns characteristic of obesity but may also contribute to increased adiposity and systemic inflammation (26, 34–38).

Understanding nutrient requirements in this population is essential, as current Dietary Reference Values (DRVs) are based on healthy, normal-weight adults (39–42). and may not accurately reflect the metabolic and physiological needs of individuals with overweight or obesity. Furthermore, much of the existing evidence relies on serum biomarkers or dietary self-reports, limiting its generalizability to the wider population (26, 28–31, 43).

To date, research on dietary adequacy in Mediterranean adults has primarily focused on overall population averages or specific clinical subgroups, with limited attention to differences by body weight status. Large national studies such as ANIBES (44), PREDIMED-Plus (45, 46), and ATTICA (47) have characterized Mediterranean dietary patterns and nutrient intakes but have not systematically compared micronutrient adequacy across BMI categories. Although associations between obesity and nutrient intake have been investigated in other contexts (48, 49), no study has jointly examined quantitative nutrient intake, Mediterranean diet adherence, and anthropometric parameters in a Spanish Mediterranean adult population.

The rationale for this study stems from the need to better understand how nutrient adequacy and dietary quality vary across BMI categories within Mediterranean populations, where diet composition and adherence to the Mediterranean Diet may influence nutritional status and metabolic health. Despite extensive research on obesity and diet, few studies have examined these relationships in Spanish adults using region-specific, validated dietary assessment tools.

To address this gap, the present study, conducted within the framework of the ongoing Tech4Diet project on 4D modeling and visualization of the human body, aimed to investigate dietary intake, nutrient adequacy, and adherence to the Mediterranean Diet across BMI categories in a Spanish Mediterranean adult population. The specific objectives were to: (1) assess compliance with the European Food Safety Authority (EFSA) Dietary Reference Values (DRVs) for macro- and micronutrients across BMI groups (42); (2) identify nutrient inadequacies associated with overweight and obesity; and (3) determine the association between adherence to the Mediterranean Diet and BMI status.

## 2 Materials and methods

### 2.1 Study design and setting

This cross-sectional observational study was conducted between September 2020 and June 2021 as part of the *Tech4Diet* project, hosted within the *Tech4D: Improving Obesity Treatment* platform (https://tech4d.ua.es/en/tech4diet.html) at the University of Alicante, Spain. All measurements and interviews were carried out at the ALINUA facility, a health center affiliated with the Faculty of Health Sciences. The study adhered to the Strengthening the Reporting of Observational Studies in Epidemiology (STROBE) guidelines for cross-sectional studies.

### 2.2 Participants

#### 2.2.1 Eligibility criteria

Participants were eligible if they were adults (≥18 years), residing in Spain, able to read and write Spanish, and willing to provide informed consent. Individuals were excluded if they were pregnant or breastfeeding; had participated in supervised dietary or nutritional treatment within the previous year; had endocrine, metabolic, or systemic disorders (e.g., thyroid, pituitary, adrenal dysfunction, diabetes, or metabolic syndrome); used chronic medication known to affect appetite, weight, or metabolism (e.g., corticosteroids, antipsychotics, thyroid hormones); had neurological conditions (e.g., stroke, Parkinson's disease); experienced a traumatic brain injury with loss of consciousness for more than 30 min; or had severe psychiatric disorders as defined by DSM-IV-TR (50) or were receiving psychiatric treatment, due to the influence of psychotropic medication on diet and metabolism.

#### 2.2.2 Recruitment and sample size

Participants were recruited through public announcements on the *Tech4D: Improving Obesity Treatment* website, which is hosted by the University of Alicante but publicly accessible. Additionally, posters were displayed in all health centers across the city of Alicante to promote the study and provide a link for individuals interested in participating. Recruitment occurred between September–November 2020 for overweight and obese participants, and June 2021 for the normal-weight group, due to pandemic-related logistical constraints.

A total of 171 individuals expressed interest, of whom four were excluded due to endocrine–metabolic disorders. The final sample comprised 167 adults (38 normal weight, 47 overweight, and 82 obese). The required sample size was determined using G*Power 3.1, a free and publicly available statistical software program for power analysis. The analysis indicated that at least 35 participants per group were needed to detect a medium effect size (*f* = 0.25) with 80% power and a significance level of α = 0.05.

### 2.3 Study variables

#### 2.3.1 Exposure and outcome variables

The main exposure variable was BMI category. Participants were categorized into three groups according to their BMI, based on WHO criteria: individuals with a BMI between 18.5 and 24.9 kg/m^2^ were classified as having normal weight; those with a BMI between 25.0 and 29.9 kg/m^2^ were classified as overweight; and individuals with a BMI equal to or greater than 30.0 kg/m^2^ were classified as having obesity. Primary outcome variables included nutrient intake, nutrient adequacy, and adherence to the Mediterranean Diet (MD). Secondary outcomes included anthropometric, clinical, and physical activity measures.

Confounders included sex, age, and physical activity, which were adjusted for in descriptive and inferential analyses.

### 2.4 Data sources and measurement

#### 2.4.1 Sociodemographic and lifestyle data

Sociodemographic data (age, sex, education level, employment status, marital status) and lifestyle information (physical activity) were collected through structured face-to-face interviews administered by a trained dietitian using a standardized questionnaire developed for the Tech4Diet project. Responses were entered into a coded database and verified for completeness by a second researcher.

#### 2.4.2 Anthropometric and clinical measurements

All anthropometric and clinical measurements were performed during a single morning session after a 12-h overnight fast, following international protocols (51). Participants were barefoot, wearing light clothing, and were instructed to avoid caffeine, alcohol, and vigorous physical activity during the fasting period.

**Anthropometry and Body Composition:** Weight and body composition, body fat and visceral fat, were measured using a TANITA MC-780MA P analyser (precision 0.1 kg). Height was measured using a SECA 213 stadiometer to the nearest 0.1 cm. Waist and hip circumferences were measured using a non-stretchable SECA 201 tape following WHO guidelines (52). Waist circumference (WC) was taken at the midpoint between the lowest rib and the iliac crest, with participants standing erect and breathing normally. Hip circumference (HC) was measured at the widest point over the buttocks. Waist-to-hip ratio (WHR) and waist-to-height ratio (WHtR) were calculated as WC/HC and WC/height (cm), respectively. WHR > 0.90 in men or > 0.85 in women and WHtR > 0.5 were considered elevated (53). A 3D optical scanner captured surface geometry for body volume estimation.

**Clinical Parameters:** Blood pressure was measured in a seated position using a calibrated OMRON monitor after 5 min of rest. Two readings were taken 1–2 min apart, and the mean value was used. Elevated blood pressure was defined as ≥130/85 mmHg (54, 55). Capillary blood samples were collected immediately afterward to measure fasting glucose, cholesterol, and triglycerides using the Accutrend^®^ Plus (Roche Diagnostics). Fasting glucose ≥110 mg/dL indicated elevated glycaemia, consistent with WHO guidelines (56).

#### 2.4.3 Physical activity assessment

Physical activity was assessed using the validated short form of the International Physical Activity Questionnaire (IPAQ-SF) (57). Frequency (days/week) and duration (minutes/day) of walking, moderate, and vigorous activities were recorded, converted to MET-min/week (3.3, 4.0, and 8.0 METs, respectively), and classified into low (< 600 MET-min/week), moderate (600–3,000), or high (>3,000) categories per IPAQ scoring guidelines.

#### 2.4.4 Dietary assessment

To analyse the spectrum of foods consumed by this sample of Mediterranean Spanish population, we used a validated food frequency questionnaire (FFQ) used in this Mediterranean region survey. FFQs are widely used for reasons of efficiency in studies that involve diet as one of the elements considered (58–61). Additionally, a 3-day 24 h food record was included, two weekdays and one weekend day to obtain a full picture of the food intake throughout the week.

Assessment of dietary intake was performed on an individual basis by a skilled clinical dietitian, who administered a 93 items FFQ to evaluate individual's diet (62). The questionnaire included nine food and beverage groups: dairy products, eggs, meat and meat products, fish, vegetables and legumes, fruits, cereals and starchy foods, fats and oils, sweets, drinks, and precooked ready meals. It also collected information on the type of fat used for cooking or dressing (e.g., olive oil, margarine), frequency of pan-fried food consumption, use of dietary supplements, and recent changes in diet. Participants reported how often they consumed each food item during two seasonal periods (summer and winter) over the previous year. Participants selected one of nine possible frequency categories for each food item, ranging from: (1) never or less than once per month; (2) one to three times per month; (3) once per week; (4) two to four times per week; (5) five to six times per week; (6) once per day; (7) two to three times per day; (8) four to five times per day; and (9) six or more times per day.

Additionally, to determine the portion sizes, a life-size photographic album was used to display different dishes, which was shown to the participant so that they could compare the portion they usually eat with the one shown in the photograph, taking those portions as the unit of measurement (63).

Frequencies of consumption were converted into daily intake values. These were multiplied by the standard portion size in grams, corrected for edible fractions and cooking weight changes, and transformed into nutrient intakes using the Mataix Verdú Spanish Food Composition Tables (64). When data were unavailable, complementary Spanish or international sources were consulted (65, 66). Nutrient data were entered and processed using custom Excel spreadsheets linked to food codes from the database.

Micronutrient and macronutrient intakes were compared to the Dietary Reference Values established by the European Food Safety Authority (EFSA) (42). Energy requirements were adjusted individually based on sex, age, and physical activity levels. Macronutrient adequacy was assessed using EFSA's Acceptable Macronutrient Distribution Ranges (AMDRs): 20–35% of energy from fats (target: 27.5%), and 45–60% from carbohydrates (target: 57.5%). Protein requirements were calculated as 0.83 g/kg of body weight per day, following EFSA and Spanish national guidelines (41, 42, 67). These thresholds represent average intake levels sufficient to meet the nutritional needs of healthy individuals by life stage and sex group.

To assess adherence to the Mediterranean Diet (MD), the Mediterranean Diet Adherence Screener (MEDAS) questionnaire was administered. This 14-item tool, developed and validated by the PREDIMED research group specifically for the Spanish population, has shown reliability in measuring MD adherence levels (68, 69). Each question in the MEDAS is scored as either +1 for responses favorable to MD guidelines or 0 for those that are not. The total adherence score is calculated by summing the individual scores across all 14 items, thereby providing an overall adherence level. Total scores ranged from 0 to 14. A score of 9 or higher was considered high adherence, while scores below 9 indicated low adherence.

### 2.5 Bias control and data quality

To minimize information bias, all measurements were taken by trained personnel following standardized procedures. Dietary data were reviewed for completeness and cross-checked with 3-day records. Implausible dietary energy intakes (< 500 or >4,000 kcal/day) were rechecked and confirmed. Anthropometric and clinical measures were double-checked for consistency.

### 2.6 Statistical analysis

Statistical analyses were conducted using IBM^®^ SPSS^®^ Statistics v27 (IBM Corp., Armonk, NY, USA) and Python 3.6 (Python Software Foundation, Beaverton, OR, USA). Descriptive statistics were used to summarize the sample characteristics. Continuous variables were expressed as mean ± standard deviation (SD) for normally distributed data. Categorical variables were presented as frequencies and percentages.

Normality of continuous variables was assessed using the Shapiro–Wilk test, and homogeneity of variances was evaluated with Levene's test. Between-group comparisons were performed using one-way analysis of variance (ANOVA) or the Kruskal–Wallis test, followed by Tukey's HSD or Bonferroni-adjusted *post-hoc* tests as appropriate. Associations between categorical variables were analyzed using the Chi-square test with Cramer's V to assess effect strength. For multiple micronutrient comparisons, the Benjamini–Hochberg false discovery rate (FDR) procedure (*q* = 0.05) was applied to adjust *p*-values. Effect sizes (partial η^2^, ϵ^2^, and Cohen's *d*) with 95% confidence intervals were reported to complement significance testing.

A multiple linear regression model was used to examine the association between adherence to the Mediterranean diet and BMI. The model included age, sex, education level, employment status, physical activity category and total energy intake as covariates. Before running the main model, assumptions of linearity, independence and homoscedasticity were assessed. Homoscedasticity was evaluated using the White test, with no evidence of heteroscedasticity detected; therefore, standard ordinary least squares estimates were retained. Multicollinearity was examined through variance inflation factors (VIF), which were all below 1.20, indicating no collinearity concerns. Regression coefficients (B), standard errors, confidence intervals and *p*-values were reported.

### 2.7 Ethical considerations

The study was approved by the Ethics Committees of the University of Alicante and ISABIAL (reference CEIm: 180380) and conducted in accordance with the Declaration of Helsinki. All participants provided written informed consent. The research was supported by the Spanish Ministry of Science and Innovation through projects TIN2017-89069-R and PID2020-119144RB100.

## 3 Results

### 3.1 Sociodemographic characteristics

Sociodemographic data for the 167 participants are summarized in Table 1. The sample included both male and female adults, with no statistically significant differences in sex distribution across BMI categories (*p* = 0.689). The participants' mean age was 46.3 ± 9.5 years (range = 22–67 years), with no significant differences in age distribution across BMI categories (*p* = 0.653).

**Table 1:** Sociodemographic characteristics by BMI category.

**Variable**	**Category**	**Normal weight**	**Overweight**	**Obese**	***p*-value**
		**(*n* = 38)**	**(*n* = 47)**	**(*n* = 82)**	
Sex	Male	17 (25.8%)	19 (28.8%)	30 (45.5%)	0.689
	Female	21 (20.8%)	28 (27.7%)	52 (51.5%)	
Age (years)	Mean (SD)	42.84 (9.64)	46.38 (9.59)	47.93 (8.98)	0.653
Marital status	Single	8 (25.8%)	11 (35.5%)	12 (38.7%)	0.546
	Married	25 (21.7%)	33 (28.7%)	57 (49.6%)	
	Divorced	5 (26.3%)	3 (15.8%)	11 (57.9%)	
	Other	0 (0.0%)	0 (0.0%)	2 (100%)	
Employment status	Employed (FT/PT)	36 (24.3%)	45 (30.4%)	67 (45.3%)	0.073
	Unemployed/other	2 (13.3%)	2 (13.3%)	15 (75.7%)	
Educational level	No studies / primary	2 (13.3%)	4 (26.7%)	9 (60.0%)	0.007
	Secondary	11 (18.0%)	10 (16.4%)	40 (65.6%)	
	University	25 (27.5%)	33 (36.3%)	33 (36.3%)	

Values are shown as frequency (percentage). FT, Full-time; PT, Part-time; SD, Standard Deviation.

Most sociodemographic variables, including age and employment status, did not differ significantly between groups. However, educational level showed a statistically significant variation across BMI categories (*p* = 0.007).

Among participants with normal weight, 13.3% had only primary education or no formal education, 18.0% had completed secondary education, and 27.5% held a university degree. In contrast, participants with overweight and obesity showed higher proportions of secondary education (16.4% and 65.6%, respectively) and similar rates of university education (both 36.3%).

### 3.2 Anthropometric and clinical characteristics

As shown in Table 2, significant differences were observed across BMI categories for most anthropometric, clinical, and dietary variables. Large effect sizes (η^2^>0.14) were found for all body composition indicators, including waist circumference, hip circumference, waist-to-hip ratio, body fat percentage, and visceral fat rating (*p* < 0.001 for all). Mean waist circumference increased from 75.3 cm in the normal-weight group to 109.4 cm in participants with obesity, while visceral fat ratings rose sharply from 3.98 to 12.95, reflecting substantial changes in central adiposity.

**Table 2:** Anthropometric, clinical, and dietary characteristics of the sample by BMI category.

**Variable**	**Normal weight**	**Overweight**	**Obesity**	***p*-value**	**η^2^**	**Interpretation**
	**(*n* = 38)**	**(*n* = 47)**	**(*n* = 82)**	
Waist circumference (cm)	75.33 ± 7.21	92.86 ± 7.34	109.37 ± 12.95	< 0.001	0.63	Large
Hip circumference (cm)	95.13 ± 5.08	105.74 ± 6.23	120.39 ± 9.26	< 0.001	0.65	Large
Waist-to-hip ratio (WHR)	0.79 ± 0.05^a^	0.88 ± 0.06	0.91 ± 0.10	< 0.001	0.56	Large
Body fat (%)	30.19 ± 9.01^a^	39.09 ± 10.10	39.27 ± 6.24	< 0.001	0.56	Large
Visceral fat (rating)	3.98 ± 2.49^a^	8.95 ± 3.71	12.95 ± 3.60	< 0.001	0.46	Large
Glucose (mg/dL)	79.08 ± 21.13	82.40 ± 19.09	87.96 ± 27.19	0.138	0.03	Small
Cholesterol (mg/dL)	198.74 ± 40.35	212.65 ± 45.00	205.70 ± 32.69	0.240	0.02	Small
Triglycerides (mg/dL)	202.63 ± 37.45	215.23 ± 40.15	211.15 ± 50.01	0.408	0.01	Very small
Systolic blood pressure (mmHg)	110.69 ± 15.38^a^	126.91 ± 17.39	125.53 ± 12.85	< 0.001	0.19	Large
Diastolic blood pressure (mmHg)	71.71 ± 7.56^a^	80.28 ± 9.63	85.71 ± 9.85	< 0.001	0.25	Large
Mediterranean diet adherence (MEDAS)	8.74 ± 1.96^a^	7.13 ± 2.11	6.54 ± 2.24	< 0.001	0.143	Large

Values are presented as mean ± SD. SD, standard deviation; WHR, waist-to-hip ratio. Superscript^a^ indicates significant difference compared to overweight and obese groups in *post-hoc* analysis (*p* < 0.05). Effect sizes were calculated using η^2^ and interpreted as follows: very small (< 0.01), small (0.01–0.06), medium (0.06–0.14), large (>0.14).

Systolic and diastolic blood pressure also differed significantly among groups (*p* < 0.001), showing medium to large effect sizes (η^2^ = 0.19 and 0.25, respectively). *Post-hoc* comparisons indicated that individuals with normal weight had significantly lower blood pressure values than those classified as overweight or obese. In contrast, no significant differences were observed between overweight and obese participants for either measure (*p*>0.05).

Metabolic parameters such as fasting glucose, total cholesterol, and triglycerides did not vary significantly across BMI categories (*p*>0.10), with small or very small effect sizes (η^2^ < 0.03). This suggests that, in this sample, changes in adiposity were more strongly reflected in anthropometric and hemodynamic measures than in fasting biochemical indicators.

### 3.3 Cardiometabolic indicators and physical activity

Tables 2, 3 present the cardiometabolic indicators and physical activity levels across BMI categories. High blood pressure was strongly associated with BMI classification (*p* < 0.001, Cramer's V = 0.374, large effect). The prevalence of elevated blood pressure increased from 10.5% in normal-weight participants to 40.4% in those with overweight and 57.3% among individuals with obesity, indicating a clear gradient in hypertension risk with increasing adiposity.

**Table 3:** Descriptive health parameters and Mediterranean diet adherence of the sample by weight group.

**Variable**	**Category**	**Normal weight**	**Overweight**	**Obese**	***p*-value**	**Cramer's V**	**Interpretation**
		**(*n* = 38)**	**(*n* = 47)**	**(*n* = 82)**	
High blood pressure	No	34 (89.5%)	28 (59.6%)	35 (42.7%)	< 0.001	0.374	Large
	Yes	4 (10.5%)	19 (40.4%)	47 (57.3%)			
Physical activity	Low	2 (5.3%)	13 (27.7%)	50 (61.1%)	< 0.001	0.290	Moderate
	Moderate	6 (14.6%)	11 (23.4%)	14 (17.1%)			
	High	30 (80.0%)	23 (48.9%)	18 (21.9%)			
Waist-to-height ratio (WHtR)	≤ 0.5	31 (81.6%)	11 (23.4%)	1 (1.2%)	< 0.001	0.725	Very large
	>0.5	7 (18.4%)	36 (76.6%)	81 (98.8%)			
High fasting blood glucose	No	37 (97.3%)	44 (93.6%)	71 (86.6%)	0.012	0.159	Not significant
	Yes	1 (2.7%)	3 (6.4%)	11 (13.3%)			
Mediterranean Diet adherence	No	24 (63.2%)	42 (89.4%)	72 (87.8%)	0.001	0.280	Moderate
	Yes	14 (36.8%)	5 (10.6%)	10 (12.2%)			

Values are shown as frequency (percentage). WHtR, waist-to-height ratio. Significant differences based on Chi-square tests at *p* < 0.05. Effect sizes were calculated using Cramer's V and interpreted as: 0–0.10 = Very small/Not significant, 0.11–0.30 = Moderate, 0.31–0.50 = Large, >0.50 = Very large.

For Mediterranean diet adherence: “Yes” indicates participants scoring ≥9 on the MEDAS questionnaire (high adherence), and “No” indicates participants scoring < 9 (low adherence).

Waist-to-height ratio (WHtR), a recognized marker of cardiometabolic risk, showed a very large effect size (*V* = 0.725). Nearly all participants with obesity (98.8%) had WHtR values greater than 0.5, compared with 76.6% of overweight and only 18.4% of normal-weight participants. These results confirm WHtR's strong predictive value for central adiposity and related health risks (52, 53).

Fasting glucose levels displayed a weaker association with BMI (*p* = 0.012, Cramer's V = 0.159). Elevated fasting glucose (≥110 mg/dL) was observed in 13.3% of obese participants, compared with 6.4% of those overweight and 2.7% of normal-weight individuals. Although the overall effect size was small, the pattern suggests an increased likelihood of impaired glucose regulation with higher BMI.

Physical activity level, assessed using the IPAQ short form, differed significantly across groups (*p* < 0.001, Cramer's V = 0.290, moderate effect). Among normal-weight participants, 80.0% reported high physical activity, compared with 48.9% in the overweight and only 21.9% in the obese group. Conversely, low activity levels were reported by 61.1% of participants with obesity. These findings align with the inverse association between physical inactivity and obesity risk.

Together, these results indicate that higher BMI is consistently linked with increased cardiometabolic risk and less favorable lifestyle behaviors, including lower physical activity.

### 3.4 Adherence to the Mediterranean diet

Adherence to the Mediterranean Diet (MD), as measured by the MEDAS score, differed significantly across BMI categories (*p* < 0.001). As shown in Table 2, mean MEDAS scores were highest among normal-weight participants (8.74 ± 1.96), followed by those who were overweight (7.13 ± 2.11) and those with obesity (6.54 ± 2.24), indicating progressively lower adherence with increasing BMI. Participants were classified as having high MD adherence if they scored 9 or above on the MEDAS questionnaire.

Among those with high adherence (*n* = 29), 48.3% were in the normal-weight group, 17.2% were overweight, and 34.5% had obesity. Conversely, among those with low adherence (MEDAS score < 9; *n* = 138), only 17.4% were normal weight, while 30.4% and 52.2% were overweight and obese, respectively. This pattern demonstrates an inverse relationship between adherence to the Mediterranean Diet and BMI classification.

Tables 4, 5 summarize the multivariable regression model assessing factors independently associated with BMI. The model was statistically significant, *F*(7, 159) = 9.512, *p* < 0.001, explaining 29.5% of the variance in IMC (*R*^2^ = 0.295; adjusted *R*^2^ = 0.264). Adherence to the Mediterranean diet was a significant independent predictor of IMC (*B* = −0.634, SE = 0.197, *p* = 0.002), indicating that higher adherence was associated with lower IMC after adjustment for all covariates. Physical activity was also strongly associated with IMC (*B* = −4.962, *p* < 0.001), with more active individuals showing markedly lower IMC values. Higher education level was associated with lower IMC (*B* = −2.452, *p* = 0.001). Age, sex, employment status and total energy intake were not significant predictors in the adjusted model. No multicollinearity was detected, with all VIF values below 1.20.

**Table 4:** Multiple linear regression model examining predictors of BMI.

**Predictor**	**B**	**SE**	**t**	**p**	**95% CI**	**Std. β**	**VIF**
Constant	–3.722	8.010	–0.465	0.643	[–19.553, 12.109]	–	–
Age (years)	0.064	0.048	1.331	0.185	[–0.031, 0.159]	0.10	1.021
MEDAS (14-item)	–0.634	0.197	–3.215	0.002	[–1.023, -0.244]	–0.26	1.038
Sex	0.340	0.931	0.368	0.713	[-1.498, 2.178]	0.03	1.180
Education level	–2.452	0.724	–3.385	0.001	[–3.882, –1.022]	–0.25	1.180
Employment status	1.296	1.126	1.151	0.255	[–0.925, 3.518]	0.09	1.052
Physical activity	–4.962	1.172	–4.234	< 0.001	[–7.271, –2.654]	–0.37	1.077
Energy intake (kcal/day)	0.001	0.001	1.800	0.074	[0.000, 0.003]	0.14	1.036

B, unstandardised regression coefficient; SE, standard error; CI, confidence interval. BMI expressed in kg/m^2^. Statistical significance was set at *p* < 0.05. All categorical predictors were dummy-coded according to the dataset. Standardized coefficients (β) indicate the relative strength of each predictor. VIF values < 2 indicate absence of multicollinearity.

**Table 5:** Summary of model fit indices, diagnostic tests, and assumption checks for the multiple linear regression predicting BMI.

**Statistic / diagnostic**	**Value**
Model significance (ANOVA)	*F*(7, 159) = 9.512, *p* < 0.001
R^2^	0.295
Adjusted *R*^2^	0.264
White test for heteroscedasticity	Non-significant
Multicollinearity (VIF)	1.021–1.180
Strongest predictor	Physical activity (β = −0.37)
Mediterranean diet effect	*B* = −0.634, *p* = 0.002
Secondary significant predictor	Education level (*B* = −2.452, *p* = 0.001)
Non-significant covariates	Age, sex, employment, energy intake

Taken together, these results indicate that greater adherence to the Mediterranean Diet, higher education level, and higher physical activity are independently linked to lower BMI, supporting the combined influence of diet quality, lifestyle, and sociodemographic factors on body weight within this Mediterranean population.

### 3.5 Nutrient intake

#### 3.5.1 Descriptive nutrient intake by BMI category

Table 6 presents the mean daily intake of energy, macronutrients, and micronutrients across BMI-defined groups. No significant differences were found in total energy intake among normal-weight, overweight, and obese participants (*p* = 0.15). However, several dietary components showed unadjusted differences across BMI categories. Protein and fiber intake were lower in the obese group compared with overweight or normal-weight participants (Kruskal–Wallis *p* = 0.010 and *p* = 0.024, respectively), with Bonferroni-adjusted pairwise tests confirming lower values for the obese group.

**Table 6:** Mean daily intake of energy, macronutrients, and micronutrients by BMI category.

**Variable**	**All**	**Normal weight**	**95% CI**	**Overweight**	**95% CI**	**Obese**	**95% CI**	***p*-value (KW)**	***p*-value FDR**	**ϵ^2^**	**Interpretation**
Energy (kcal)	2388.54 ± 774.92	2303.78 ± 783.04	2046.40–2561.16	2523.31 ± 743.86	2304.90–2741.72	2350.57 ± 788.02	2177.42–2523.72	0.15	0.31	0.01	Very small
Protein (g)	113.04 ± 43.00	105.35 ± 37.32	93.08–117.62	123.02 ± 33.96	113.05–132.99	110.88 ± 49.13	100.08–121.68	0.01	0.21	0.04	Small
Lipids (g)	98.79 ± 39.34	95.81 ± 34.51	84.47–107.15	101.09 ± 32.17	91.64–110.54	98.85 ± 45.08	88.94–108.76	0.48	0.57	0.00	Very small
Carbohydrates (g)	266.09 ± 93.05	259.85 ± 111.55	223.18–296.52	283.71 ± 99.14	254.60–312.82	258.89 ± 78.95	241.54–276.24	0.24	0.31	0.01	Very small
Fiber (g)	36.09 ± 16.53	38.72 ± 19.03	32.46–44.98	40.44 ± 17.95	35.17–45.71	32.39 ± 13.54	29.41–35.37	0.02	0.14	0.03	Small
Sodium (mg)	3792.19 ± 1410.98	3405.07 ± 1287.22	2981.97–3828.17	4096.90 ± 1343.62	3702.40–4491.40	3796.93 ± 1474.35	3472.98–4120.88	0.07	0.21	0.02	Small
Potassium (mg)	4585.34 ± 2022.71	5002.22 ± 2304.85	4244.63–5759.81	4795.91 ± 2026.14	4201.01–5390.81	4190.22 ± 1754.15	3804.79–4575.65	0.04	0.18	0.03	Small
Calcium (mg)	1241.93 ± 640.44	1181.54 ± 544.77	1002.48–1360.60	1345.38 ± 507.50	1196.37–1494.39	1210.63 ± 741.51	1047.70–1373.56	0.07	0.21	0.02	Small
Magnesium (mg)	558.47 ± 224.76	527.93 ± 231.00	452.00–603.86	606.65 ± 220.85	541.81–671.49	545.01 ± 222.45	496.13–593.89	0.11	0.26	0.02	Small
Iron (mg)	23.79 ± 9.39	21.04 ± 9.17	18.03–24.05	24.85 ± 9.12	22.17–27.53	23.59 ± 9.72	21.45–25.73	0.14	0.28	0.01	Small
Zinc (mg)	11.62 ± 4.31	11.36 ± 4.03	10.04–12.68	12.66 ± 3.56	11.61–13.71	11.14 ± 4.74	10.10–12.18	0.01	0.21	0.04	Small
Selenium (μg)	86.16 ± 33.53	76.82 ± 28.83	67.34–86.30	94.26 ± 31.47	85.02–103.50	85.84 ± 35.82	77.97–93.71	0.01	0.21	0.04	Small
Iodine (μg)	122.74 ± 70.00	114.37 ± 43.86	99.95–128.79	137.78 ± 46.12	124.24–151.32	117.99 ± 53.39	106.26–129.72	0.02	0.14	0.04	Small
Vitamin B1 (mg)	1.93 ± 0.74	1.91 ± 0.70	1.68–2.14	2.15 ± 0.79	1.92–2.38	1.82 ± 0.72	1.66–1.98	0.03	0.18	0.03	Small
Vitamin B2 (mg)	2.99 ± 1.98	2.74 ± 1.38	2.29–3.19	3.27 ± 2.27	2.60–3.94	2.93 ± 2.03	2.48–3.38	0.33	0.57	0.00	Very small
Vitamin B6 (mg)	3.26 ± 1.43	2.99 ± 1.26	2.58–3.40	3.61 ± 1.63	3.13–4.09	3.14 ± 1.44	2.82–3.46	0.04	0.18	0.03	Small
Vitamin B12 (μg)	12.02 ± 10.21	7.92 ± 1.91	7.29–8.55	11.91 ± 2.88	11.06–12.76	13.02 ± 3.41	12.27–13.77	0.82	0.82	0.00	Very small
Folate (μg)	514.01 ± 260.47	565.19 ± 262.08	479.05–651.33	577.95 ± 269.86	498.72–657.18	453.64 ± 242.94	400.26–507.02	0.01	0.21	0.05	Small
Vitamin C (μg)	268.87 ± 168.52	300.91 ± 168.45	245.54–356.28	310.55 ± 162.86	262.73–358.37	228.74 ± 140.07	197.96–259.52	0.01	0.21	0.05	Small
Vitamin A (μg)	1829.50 ± 1103.50	2013.69 ± 377.64	1889.56–2137.82	2031.55 ± 1241.66	1666.98–2396.12	1628.35 ± 1050.17	1397.60–1859.10	0.02	0.14	0.04	Small
Vitamin D (μg)	7.41 ± 4.39	8.23 ± 5.29	6.49–9.97	7.43 ± 3.99	6.26–8.60	7.02 ± 4.13	6.11–7.93	0.77	0.82	0.00	Very small
Vitamin E (mg)	16.78 ± 7.62	19.01 ± 9.16	16.00–22.02	17.56 ± 6.52	15.65–19.47	15.69 ± 6.00	14.37–17.01	0.03	0.18	0.03	Small
Omega-6 (%)	23.82 ± 18.73	20.06 ± 11.85	16.17–23.95	22.95 ± 13.90	18.87–27.03	25.66 ± 23.08	20.59–30.73	0.22	0.28	0.01	Very small
Omega-3 (%)	2.62 ± 1.07	2.60 ± 1.17	2.22–2.98	2.66 ± 0.95	2.38–2.94	2.60 ± 1.10	2.36–2.84	0.77	0.82	0.00	Very small

Values are presented as mean ± SD. KW, Kruskal–Wallis test; FDR, false discovery rate adjusted using the Benjamini–Hochberg method. Effect size ε^2^ is shown with interpretation: 0–0.10 = Very small/Not significant, 0.11–0.30 = Small, 0.31–0.50 = Medium, >0.50 = Large/Very large. Units: kcal, kilocalories/day; g, grams/day; mg, milligrams/day; μg, micrograms/day; %, percentage of total energy.

Unadjusted analyses also revealed between-group variation in several micronutrients, including zinc (*p* = 0.011), potassium (*p* = 0.040), selenium (*p* = 0.014), and iodine (*p* = 0.015), as well as in vitamin B1, vitamin B6, folate, vitamin C, vitamin A, and vitamin E (*p* < 0.05 for all). However, after applying the Benjamini–Hochberg false discovery rate (FDR) correction (*q* = 0.05) for multiple comparisons, none of these differences remained statistically significant (FDR-adjusted *p*>0.05).

Although statistical significance was lost after adjustment, small effect sizes (ε^2^ = 0.03–0.05) were observed for several nutrients (protein, fiber, potassium, zinc, selenium, iodine, vitamins A, B1, B6, C, E, and folate), suggesting modest yet consistent differences in nutrient intake by BMI category. These findings indicate that, despite comparable total energy intake, individuals with obesity may consume diets of lower nutrient density, underscoring the potential importance of food quality over quantity in weight management.

#### 3.5.2 Compliance with recommended daily intake of energy, vitamins, and minerals

Table 7 presents overall compliance with DRVs and compares these values across BMI categories. Mean energy intake exceeded recommendations (108.07%), yet 45.51% of the full sample consumed less than the recommended levels. This imbalance was most marked in the normal-weight group, where 55.26% of individuals fell below requirements, compared with 38.30% of the overweight group and 45.12% of those with obesity.

**Table 7:** Compliance with recommended daily intake of energy, macronutrients, and micronutrients by BMI category.

**Nutrient**	**All (Mean ±SD)**	**Below all (%)**	**Normal Weight**	**Below NW (%)**	**Overweight**	**Below OW**	**Obese**	**Below OB**
						**(%)**		**(%)**
Energy (kcal)	108.07 ± 37.81	45.51%	101.26 ± 34.52	55.26%	114.05 ± 37.66	38.30%	107.79 ± 39.21	45.12%
Protein (g)	168.75 ± 70.64	16.77%	203.41 ± 67.82	5.26%	195.41 ± 60.80	6.38%	137.42 ± 63.68	28.05%
Lipids (g)	146.44 ± 61.71	17.96%	138.11 ± 49.24	15.79%	149.50 ± 52.40	17.02%	148.54 ± 71.38	19.51%
Carbohydrates (g)	91.72 ± 34.63	70.06%	87.15 ± 39.31	78.95%	97.80 ± 37.99	65.96%	90.35 ± 30.00	68.29%
Fiber (g)	144.37 ± 66.11	26.35%	154.90 ± 76.11	26.32%	161.74 ± 71.80	19.15%	129.54 ± 54.17	30.49%
Sodium (mg)	189.61 ± 70.55	8.38%	170.25 ± 64.36	15.79%	204.84 ± 67.18	4.26%	189.85 ± 73.72	7.32%
Potassium (mg)	131.01 ± 57.79	32.93%	142.92 ± 69.81	26.32%	144.27 ± 61.20	25.53%	117.89 ± 46.35	40.24%
Calcium (mg)	130.73 ± 67.41	32.93%	124.37 ± 57.34	36.84%	141.62 ± 53.42	21.28%	127.43 ± 78.05	37.80%
Magnesium (mg)	176.01 ± 73.61	13.17%	163.75 ± 70.35	18.42%	190.55 ± 73.25	4.26%	173.36 ± 74.82	15.85%
Iron (mg)	177.89 ± 77.58	8.98%	176.96 ± 88.50	15.79%	194.63 ± 78.45	6.38%	168.72 ± 70.80	7.32%
Zinc (mg)	115.02 ± 45.85	38.92%	110.02 ± 37.80	36.84%	124.72 ± 38.57	23.40%	111.79 ± 52.28	48.78%
Selenium (μg)	123.09 ± 47.90	32.34%	109.74 ± 41.18	44.74%	134.66 ± 44.96	19.15%	122.63 ± 51.17	34.15%
Iodine (μg)	81.82 ± 33.34	74.85%	76.25 ± 29.24	84.21%	91.86 ± 30.75	61.70%	78.66 ± 35.59	78.05%
Vitamin B1 (mg)	201.62 ± 83.40	5.99%	194.13 ± 70.83	2.63%	224.07 ± 91.02	4.26%	192.23 ± 82.77	8.54%
Vitamin B2 (mg)	186.96 ± 123.44	20.96%	171.03 ± 86.25	23.68%	203.84 ± 141.86	10.64%	184.66 ± 126.93	25.61%
Vitamin B6 (mg)	199.30 ± 88.47	5.99%	200.23 ± 76.79	7.89%	221.31 ± 92.86	2.13%	186.25 ± 89.43	7.32%
Vitamin B12 (μg)	300.44 ± 155.23	2.99%	319.87 ± 197.94	7.89%	297.76 ± 121.60	2.13%	292.96 ± 151.16	1.22%
Folate (μg)	155.76 ± 78.93	22.75%	171.27 ± 79.42	21.05%	175.14 ± 81.77	10.64%	137.47 ± 73.62	30.49%
Vitamin C (μg)	268.79 ± 171.66	11.38%	297.10 ± 169.59	7.89%	311.15 ± 199.45	4.26%	231.39 ± 147.76	17.07%
Vitamin A (μg)	267.57 ± 165.10	9.58%	288.49 ± 132.55	5.26%	296.31 ± 187.71	4.26%	241.39 ± 162.64	14.63%
Vitamin D (μg)	49.41 ± 29.24	96.41%	54.87 ± 35.29	97.37%	49.55 ± 26.63	93.62%	46.80 ± 27.55	97.56%
Vitamin E (mg)	143.48 ± 65.54	26.35%	160.32 ± 75.18	18.42%	150.80 ± 64.65	25.53%	131.48 ± 59.49	30.49%
Omega-6 (%)	241.48 ± 187.91	9.58%	194.38 ± 102.11	7.89%	234.05 ± 148.15	17.02%	267.56 ± 231.03	6.10%
Omega-3 (%)	213.06 ± 90.59	6.59%	203.53 ± 88.04	5.26%	217.00 ± 83.14	6.38%	215.22 ± 96.41	7.32%

Values are expressed as mean % of DRI ± SD and percentage of individuals below recommendations. DRI, Dietary Reference Intake. Units: kcal, kilocalories/day; g, grams/day; mg, milligrams/day; μg, micrograms/day. “Below” columns refer to the percentage of individuals whose intake was below the recommended levels. NW, Normal weight; OW, Overweight; OB, Obesity.

For macronutrients, the total sample showed moderate rates of inadequacy for protein (16.77%) and fiber (26.35%). These proportions increased within the obesity group, where 28.05% did not meet protein recommendations and 30.49% fell below fiber intake guidelines, compared with lower inadequacy figures in the normal-weight and overweight groups.

Micronutrient inadequacy was widespread across the full sample, particularly for iodine (74.85%) and vitamin D (96.41%). These deficiencies were evident across all BMI strata, although iodine inadequacy ranged from 61.70% in overweight individuals to 84.21% in normal-weight participants and 78.05% in those with obesity. Vitamin D inadequacy remained uniformly high across all categories, but was highest among individuals with obesity, where more than 97% did not meet recommended intakes.

Additional micronutrients showed a gradient of worsening adequacy with increasing BMI. For example, potassium inadequacy (32.93% in the full sample) reached 40.24% among individuals with obesity. Zinc inadequacy was also more common in this group (48.78%) compared with the total sample (38.92%). A similar pattern was observed for folate, where inadequacy increased from 21.05% in normal-weight participants to 30.49% in those with obesity. Vitamins A, C, and E showed higher inadequacy rates in individuals with obesity than in the population as a whole.

In contrast, overweight individuals exhibited relatively better adequacy for some nutrients. Calcium inadequacy affected only 21.28% of this group compared with 32.93% of the full sample, and magnesium inadequacy was also lowest among those overweight (4.26% vs. 13.17% overall).

Across all BMI categories, intake of omega-3 and omega-6 fatty acids exceeded recommendations for most participants, with inadequacy rates below 10% and minimal variation between groups.

Overall, data indicates that while certain inadequacies, particularly vitamin D and iodine, are pervasive, many micronutrient deficiencies become more pronounced with increasing BMI. These findings illustrate a pattern of high energy intake coexisting with suboptimal micronutrient intake, especially among individuals with obesity.

## 4 Discussion

This study examined dietary intake, nutrient adequacy, and adherence to the Mediterranean Diet in adults from a Spanish Mediterranean population, stratified by BMI category. Total energy intake did not differ significantly between normal-weight, overweight, and obese participants, although all groups exceeded EFSA energy recommendations on average. Obese individuals reported lower intakes of several key nutrients, particularly fiber, potassium, zinc, folate, and vitamins A, B1, C, and E, compared with normal-weight participants, suggesting potential differences in dietary quality across BMI categories.

Compared to other investigations, the present sample showed no important differences between overweight and obese individuals regarding adherence to recommended intakes. This discrepancy may reflect underreporting among individuals with obesity, a well-known bias influenced by social desirability and weight stigma (70), the inclusion of misreporters in dietary analyses (71), and methodological limits of self-reported energy intake (72). Similar patterns were observed across most nutrients, with overweight participants consistently reporting higher intakes than those with obesity. Discrepancies between reported and recommended nutrient intakes have also been identified in other European cohorts (73). Evidence from clinical samples indicates that obese individuals underreport energy intake more often than normal-weight adults, although differences diminish when weight is stable or attitudes toward dieting are considered (74, 75). Studies in medically supervised populations have also documented inconsistencies in reported intake among individuals with obesity, underscoring the need for caution when interpreting self-reported dietary data (76).

The analysis of a broad set of nutrients was designed to capture overall dietary patterns, and all comparisons were adjusted for multiple testing using the Benjamini–Hochberg FDR procedure. As a result, the observed associations should be interpreted as exploratory but statistically robust, providing hypotheses for future confirmatory research.

Vitamin D and iodine intake were markedly inadequate across all BMI groups, with over 96% of participants below recommended levels for vitamin D and 74% for iodine. This mirrors European data showing widespread vitamin D insufficiency, including in Mediterranean regions (77). Adults with obesity often have lower serum 25(OH)D despite adequate or high energy intake (26). Low vitamin D status has also been associated with inflammation, insulin resistance, and increased cardiometabolic risk (78). Broader evidence from European cohorts further indicates that micronutrient inadequacies, particularly of vitamin D, are common even in energy-sufficient diets (79). These findings highlight ongoing public health concerns regarding micronutrient adequacy among adults with excess body weight in Mediterranean settings.

Adherence to the Mediterranean Diet was significantly associated with BMI classification, with normal-weight participants reporting the highest scores. The multivariable regression model showed that higher MD adherence was independently associated with lower BMI. Educational level also showed a significant inverse association with BMI, supporting previous evidence that higher education may influence nutritional awareness, food choices, and overall lifestyle behaviors (80, 81). Physical activity shows a clear association with weight status. Our findings reveal a statistically significant relationship between physical activity levels and weight classification (p < 0.001), indicating that lower physical activity is consistently linked to higher weight categories. This support previous research that insufficient physical activity may contribute to energy imbalance and adiposity, reinforcing the importance of promoting active lifestyles in public health strategies aimed at obesity prevention (82, 83). In contrast, sex, employment status, age, and energy intake were not significant predictors. These findings align with large-scale evidence showing that Mediterranean-style dietary patterns are associated with healthier weight and metabolic outcomes. Several studies have shown that MD adherence is inversely related to BMI (84, 85). Consistent associations between MD adherence, healthier metabolic profiles, and reduced obesity risk have been reported across observational and interventional studies (86, 87). Other works have linked MD adherence to improved weight and nutrient profiles in Mediterranean adults (45, 88), and these benefits appear stronger when combined with regular physical activity (89).

Certain methodological considerations should be noted when interpreting these findings. The cross-sectional design restricts causal inference, but it remains appropriate for identifying associations and generating hypotheses to guide future research. The use of validated dietary assessment tools, administered by a qualified dietitian, helped ensure data quality, although, as in all studies based on self-reported intake, some degree of recall or reporting bias cannot be completely excluded. Recruitment through university-affiliated and online channels may have resulted in a sample with somewhat higher education or health awareness; however, broad inclusion criteria and community-level dissemination helped achieve participant diversity. Data collection took place in different seasons, which may have influenced food availability, though this effect was minimized by the FFQ's design, which captures annual consumption across summer and winter periods. Finally, while the sample size was modest, it was sufficient for detecting medium effect sizes and for meeting the study's exploratory objectives within a Mediterranean adult population.

Despite these limitations, the findings offer valuable insight into the relationship between BMI, nutrient adequacy, and adherence to the Mediterranean Diet. Although total energy intake was similar across groups, qualitative dietary differences emerged, indicating that nutrient density rather than caloric quantity may play a more important role in weight regulation. The strong and independent associations of MD adherence, educational attainment, and physical activity with BMI suggest that both lifestyle and socioeconomic factors contribute to dietary quality and weight status. These findings reinforce the view that public health strategies should integrate nutrition education with initiatives promoting active lifestyles.

The widespread inadequacy of vitamin D and iodine intake highlights the need for targeted interventions addressing micronutrient insufficiencies in Mediterranean populations. However, the associations observed here should be considered preliminary, serving as a foundation for hypothesis generation. Future longitudinal and interventional research is required to confirm causal pathways and to evaluate whether improving adherence to the Mediterranean Diet or correcting micronutrient deficiencies has measurable effects on obesity prevention and metabolic health.

The study sample, while regionally focused and not nationally representative, reflects dietary behaviors characteristic of Mediterranean adults and thus provides meaningful insight into diet–weight relationships within this context. The use of validated questionnaires and standardized procedures enhances the comparability and reproducibility of results with other Mediterranean-based studies. Although caution is warranted when extrapolating findings beyond this regional setting due to cultural and dietary variations, the study offers a valuable contribution to understanding how Mediterranean dietary patterns relate to nutritional adequacy and body composition.

Future research involving larger and multi-center cohorts would allow assessment of these associations across broader demographic and geographic contexts. Nonetheless, the present exploratory analysis adds to the evidence linking adherence to the Mediterranean Diet and sufficient nutrient intake with healthier weight profiles, underscoring the combined influence of diet quality, physical activity, and education as modifiable factors in metabolic health.

## 5 Conclusions

Although our sample did not show statistically significant differences between BMI groups in meeting EFSA nutritional recommendations after application of the Benjamini–Hochberg false discovery rate procedure, it remains essential to assess the nutritional quality of the diet rather than focusing solely on total energy intake. In this study, vitamin D and iodine were the most consistently inadequate nutrients across all BMI categories, with deficiencies most pronounced among individuals with obesity. The analysis of DRV compliance also showed that micronutrient inadequacy was widespread and, for several nutrients, tended to worsen with increasing BMI. While vitamin D and iodine inadequacy affected nearly all participants, other nutrients, including potassium, zinc, folate, and vitamins A, C, and E, showed higher rates of inadequacy among those with obesity. These findings indicate that high energy intake can coexist with poorer micronutrient profiles, particularly in the obese group. Overall, these results underscore the need to prioritize nutrient density and dietary quality in nutritional guidance and to monitor key micronutrients more closely in population-based strategies

In this sample, higher adherence to the Mediterranean Diet was observed among normal-weight participants. This association remained significant after adjustment for major covariates and aligns with previous longitudinal and interventional studies linking Mediterranean dietary patterns to healthier weight trajectories and improved metabolic outcomes. However, given the cross-sectional nature of the data, these associations must be interpreted as descriptive rather than causal. Further prospective and experimental studies are needed to clarify whether Mediterranean Diet adherence directly influences obesity risk.

As previously noted by other authors (90), current dietary recommendations are largely derived from healthy, normal-weight populations and may not fully account for the physiological and metabolic needs of individuals with overweight or obesity. The present findings provide exploratory support for this perspective, showing differences in nutrient adequacy and dietary quality across BMI categories. These results contribute to understanding diet–weight relationships in Mediterranean populations, and they reinforce the need for continued research to develop more tailored and evidence-based nutritional recommendations for diverse population groups.

## Data Availability

The datasets presented in this article are not readily available because the approval by the ethics committees do not allow the sharing of the datasets. Requests to access the datasets should be directed to ana.zaragoza@ua.es.
